# Clinicopathological Characteristics and Her-2/neu Status in Chinese Patients with Uterine Papillary Serous Carcinoma

**DOI:** 10.5402/2011/575327

**Published:** 2010-10-20

**Authors:** Yulan Ren, Huaying Wang, Xiaoyan Zhou, Wentao Yang, Xiaowei Huang, Yongming Lu, Daren Shi

**Affiliations:** ^1^Department of Gynecologic Oncology, Fudan University Shanghai Cancer Center, China; ^2^Department of Oncology, Shanghai Medical College, Fudan University, Shanghai 200032, China; ^3^Department of Pathology, Fudan University Shanghai Cancer Center, China

## Abstract

*Objective*. To analyze clinico-pathological features of Chinese patients with UPSC, and investigate roles of Her-2/neu protein expression and gene amplification in UPSC prognosis. *Methods*. Thirty-six patients with UPSC treated in Cancer Hospital of Fudan University from 1996 to 2006 were analysed retrospectively. Chromogenic in situ hybridization (CISH) and immunohistochemistry (IHC) were performed to evaluate Her-2/neu gene amplification and protein expression respectively. *Results*. The median age was 63 years, and 61% (22/36) were late stages (stage III/IV). The 1-year, 3-year, and 5-year overall survival (OS) was 73.1%, 51.9% and 43.9%, respectively. Advanced stages (*P* = .0006) and deep myometrial invasion (*P* = .0138) were significantly associtated with a shorter OS. In 36 cases, 27.8% (10/36) showed 2+ staining and 8.3% (3/36) showed 3+ by IHC. Amplification of the Her-2/neu gene was observed in 11.1% (4/36) cases. The 5-year overall survival rate in Her-2/neu IHC 2 + ∼3+ and 0 ~ 1+ cases was 12.9% and 68.6% respectively. Her-2/neu protein expression 2 + ∼3+ was significantly associated with advanced surgical stage and worse overall survival (*P* = .03 and *P* = .0023, resp.). *Conclusion*. Chinese patients with UPSC showed characteristics of deep myometrial invasion, advanced stages and poor overall survival. Her-2/neu protein overexpression is associated with advanced stage and poor survival outcome.

## 1. Introduction

Endometrial cancer is the fourth most common cancer among women in the United States. According to the latest data from the National Cancer Institute, approximately 40,100 new cases and 7,470 deaths from uterine cancer are expected in 2008 [1]. In Shanghai, China, the age-adjusted incidence of endometrial cancer increased by 4.41% per year between 1973 to 1975 and 1997 to 1999 [2–4]. Endometrial cancer has been divided into two subtypes (type I and II) based on different morphological appearance and clinicalpathological characteristics [5–7]. Type I, which is endometrioid histology with relatively low-grade features and good prognoses, occurs most often in postmenopausal women and is associated with unopposed estrogen exposure. In contrast, type II endometrial cancer comprises of nonendometrial histology with an aggressive clinical course and poor prognoses.

Uterine papillary serous carcinoma (UPSC) is the most common subtype of type II endometrial cancer. It was first described by Hendrickson et al. [8] in 1982, and histologically similar to high-grade ovarian cancer. The microscopic criteria for diagnosis were described as neoplastic epithelium, characterized by serous differentiation with psammoma bodies present and predominantly papillary architecture [8]. Although UPSC represents only about 10% of endometrial cancers, they account for over 50% of recurrences and deaths caused by endometrial cancer. The 5-year overall survival (OS) for women with UPSC is only 46% for all stages [9]. Comprehensive surgical staging is recommended for all patients with UPSC, regardless of the depth of myometrial invasion of the tumor [9, 10]. Chemotherapy is recommended after the surgery, but unlike ovarian papillary serous carcinomas, UPSC is a chemoresistant disease from onset, with low response rates and a short duration of response [11, 12]. Given the overall poor prognosis of these patients, new treatment modalities are urgently needed for UPSC.

Her-2/neu is one of the most studied molecular markers in anticancer therapy. Her-2/neu, also known as c-erbB2, is a member of the epidermal growth factor receptor transmembrane receptor tyrosine kinase family [13]. 25% to 30% of breast cancers were found to have Her-2/neu overexpression, which is considered as a negative prognostic factor [14, 15] as well as a predictive marker of resistance to tamoxifen therapy, benefit from doxorubicin-based adjuvant chemotherapy, and trastuzumab (anti-Her-2 monoclonal antibody) [16–18]. Recently, a striking overexpression of the Her-2/neu in UPSC has been identified by Santin et al, with the rates ranging from 18% to 80% in different studies [19–21]. In this study, we conducted a single institutional review of 10 years summary of this rare disease, which, to our knowledge, is the largest one so far analysing the clinicopathological features, prognostic factors, and Her-2/neu status of Chinese patients with UPSC. The purpose of this study is to better understand clinicopathological features of UPSC and the relationship with Her-2/neu abnormality.

## 2. Materials and Methods

### 2.1. Patient Population and Tumor Specimens

All the slides of the patients with endometrial carcinoma who underwent surgery at Cancer hospital, Fudan University, from January 1996 to January 2006, were reviewed by two pathologists separately. If there was dispute in diagnosis between them, slides would be reviewed by another pathologist who made the final diagnosis. Altogether 457 patients with endometrial cancer were treated in our hospital during the last 10 years and 36 cases of UPSC were rediagnosed, which accounted for about 8%. Their clinical and surgical pathology reports were recorded. Patients were staged according to the 1988 FIGO surgical criteria [22]. 

Overall Survival (OS) was the primary end-point evaluated. It was calculated from the date of the surgical diagnosis. The information on survival was obtained in the medical record and death certificate. If this information was not available, the patient was censored after her last contact.

### 2.2. Immunohistochemistry (IHC)

Formalin-fixed, paraffin-embedded specimens were collected from tissue bank of Cancer hospital, Fudan University. For all cases, a routine hematoxylin and eosin slide was evaluated to ensure that the specimen evaluated had serous carcinoma. IHC was performed using c-erbB-2/Her-2/neu Ab (A0485, Dako Corp., Denmark). This antibody is a polyclonal rabbit anti-human c-erbB-2 oncoprotein. IHC staining was performed on 4-um-thick paraffin-embedded sections according to the manufacturer's instructions. Briefly, after pretreatment with 10 mM citrate buffer at pH 6.0 using a steamer, they were incubated with c-erbB-2/Her-2/neu Ab at 37°C for 2 hours, then incubated with immunoperoxidase anti-rabbit Ab (K4003, Dako Corp., Denmark) for half an hour and followed by substrate-chromogen solution( DAB). Negative controls were analyzed on slides incubated without the antibodies. Her-2/neu-expressing breast cancer was used as a positive control. The intensity of immunostaining was graded as follows: negative (0); incomplete membranous staining or complete membranous staining in less than 10% of the tumor cells (1+); moderate intensity, complete membranous staining in greater than 10% of the tumor cells (2+); or strong intensity, complete membranous staining in greater than 10% of the tumor cells (3+). Tumors with 2+ or 3+ staining were considered Her-2/neu overexpression [23, 24]. The sections were examined by light microscopy by two different pathologists without knowledge of the clinical outcome.

### 2.3. Chromogenic In Situ Hybridization (CISH)

CISH was done using Zymed SPoT-Light HER-2 CISH Kit (84-0146) according to instructions of manufacture. Sections of 3 *μ*m were cut from paraffin-embedded archival tissue from tissue bank. The sections were deparaffinized and pretreated in a microwave oven at 100°C for 15 minutes. After a brief rinsing with Tris-buffered saline (0.05 mol/L Tris/HCl saline, pH 7.4–7.6), the slides were digested with Enzyme for 5 to 10 minutes (the time for each slide was different), followed by rinsing with distilled water for 3 times, and dehydrating with graded ethanols. The digoxigenin-labelled Her-2/neu probe (Zymed) was applied onto the slides, covered with coverslips and denatured at 95°C for 5 minutes. The hybridization was performed overnight at 37°C. The slides were then washed with standard saline citrate (SSC) in room temperature briefly then with SSC at 75°C for 5 minutes. Immunodetection was performed by the labelled avidin-biotin complex peroxidase system according to the manufacturer's instructions. Finally, sections were lightly counterstained with haematoxylin. Breast cancer with known Her-2/neu-amplification was used as a positive control. Benign endometrium was used as a negative control. Her-2/neu gene signals were scored in histological sections, in at least 150 neoplastic cells, using a conventional microscope (Olympus). Tumours were classified, depending on the number of gene copies in the nuclei, as normal (1–5 copies) and amplified (>6 copies or when large gene copy clusters were seen in at least 50%). In addition, amplified tumours were classified as low-level (6–10 copies) or high level (>10 copies).

### 2.4. Statistical Analysis

*X* ^2^ tests were performed to assess prognostic factors. Kaplan-Meier survival curves were used to estimate OS. The log-rank test was used to compare survival curves. Cox's proportional hazards regression was used to model survival with Her-2/neu IHC overexpression and clinicopathological variables typically associated with prognosis. Spearman correlation was used to analyze the relation between the results of IHC and CISH. SPSS version 11.5 software was used for all analyses.

## 3. Results

### 3.1. Clinicopathological Features of Patients

The median age of the patients was 63 years (mean 64 years, range 45~81 years). The median BWI (Body Weight Index) was 23.8 kg/m^2^ (mean 23.73, range 17.6~33.8). The main presenting symptoms were abnormal vaginal bleeding (86%, 31/36), while other 4 patients first presented with abdominal symptoms such as abdominal mass and ascites, and 1 with vaginal discharge. 16 patients (45.7%) were complicated with hypertension and 4 patients (11.4%) with diabetes. 8 cases or relatives had history of other cancers, including 1 with ovarian mucous adenocarcinoma, 2 with breast cancer, 1 with endometrial cancer, 3 with gastrointestinal cancer, and 1 with thyroid neoplasms. 

Of these 36 patients, 11 (31%) were stage I (1 IA, 6 IB, 4 IC), 3 (8%) were stage II (1 IIA, 2 IIB), 9 (25%) were stage III (4 IIIA, 1 IIIB, 4 IIIC), and 13 (36%) were stage IV (13 IVB). Altogether 39% were early stages (stage I/II) and 61% were late stages (stage III/IV). 21 tumors invaded more than half of myometrium, in which 11 invaded to serosa of the uterus. 15 tumors invaded less than half of myometrium. And only two patients had no myometrial invasion. 20 tumors (55.6%) demonstrated LVSI (Lymph-vascular space invasion). Twenty-seven tumors (75%) had overexpression of p53, while positive ER expression was only detected in 10 cases (27.8%), and PR expression in 7 cases (19.4%).

All patients underwent exploratory surgery, hysterectomy, bilateral salpingo-oophorectomy, omentectomy, pelvic lymphadenectomy, and/or para-aortic lymphadenectomy. Patients with stage IVB disease also received cytoreductive surgery. 4 patients received chemotherapy before the surgery. After the surgery, 14 patients received platinum-based chemotherapy including cisplatin, cyclophosphamide and anthracycline, or carboplatin and paclitaxel, and so forth. 4 patients received whole pelvic radiotherapy with 2 extended to the para-aortic field. 4 patients received whole pelvic radiotherapy combined with platinum-based chemotherapy. 6 patients had oral tamoxifen or progestin, and 8 patients were observed after the surgery.

### 3.2. IHC and CISH

**Figure 1:**
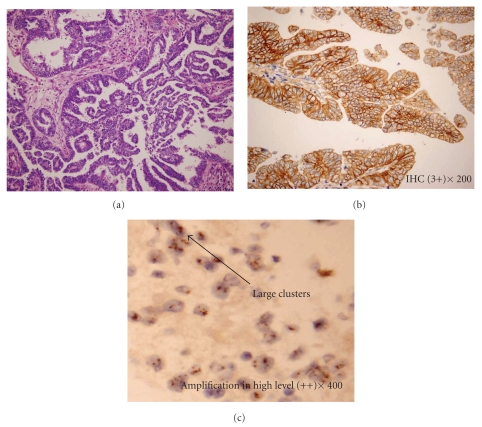
(a) Hematoxylin and eosin stain and (b) immunohistochemical staining 3+ for Her-2/neu expression on paraffin-embedded uterine serous carcinoma specimens. ×200. (c) Her-2/neu gene amplification amplified in high level. ×400.

Of the 36 cases, 13 (36.1%) patients had Her-2/neu protein overexpression by IHC. In this 13 patients, 10 (27.8%) showed 2+ and 3 (8.3%) showed 3+. (Figure 1). The remaining 11 samples showed 1+ and 12 showed 0 for Her-2/neu protein. The clinicopathological features by Her-2/neu IHC status were summarized in Table 1. Overexpression of Her-2/neu was seen in 50.0% (11/22) of UPSC patients with advanced disease (Stage III~IV), compared to 14.3% (2/14) of patients with early disease (Stage I~II). Patients with overexpression of Her-2/neu were significantly more likely to have advanced-stage disease when compared to Her-2/neu negative patients (OR = 6.0, 95%CI: 1.08–33.32, *P* = .03). 

CISH was performed in all UPSC cases. Four out of the 36 (11.1%) cases were found with Her-2/neu gene amplification, in which 3 amplified in high level and 1 in low level (Figure 1). The correlation between IHC and CISH results were assessed (Table 2). All IHC 3+ (100%, 3/3) cases had Her-2/neu gene amplification. And all IHC negative cases had normal Her-2/neu gene copies (1–5 copies). Therefore, complete concordance between IHC and CISH was observed in IHC 3+ and negative cases (*P* = .001, Spearman Correlation). Only one out of 10 cases scored as 2+ by IHC (10%, 1/10) was found with gene amplification by CISH.

### 3.3. Survival Analyses

**Figure 2:**
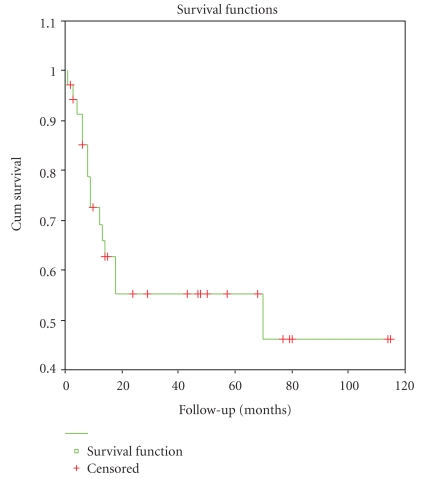
Overall survival curve for all 36 patients with UPSC.

 The median follow-up time for all of the patients was 31 months (range, < 1 to 115 months). During the follow-up time, 17 patients died (47.2%), and 15 of these deaths were related to this disease. Nine patients were observed to have disease progression during followup, with 4 in pelvis, 2 in liver, 1 in brain, 1 in both liver and pelvis, and 1 in both liver and brain. The 1-year, 3-year, and 5-year overall survival for all patients was 73.1%, 51.9%, and 43.9%, respectively (Figure 2,).

**Figure 3:**
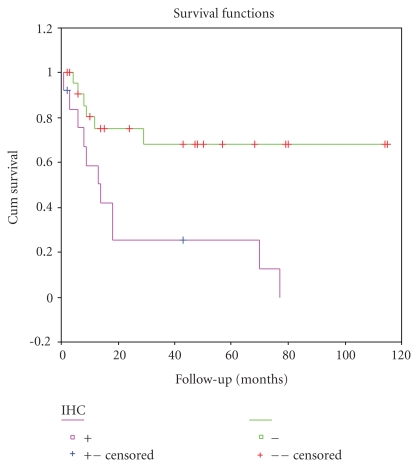
Kaplan-Meier curve based on Her-2/neu protein expression (IHC positive versus IHC negative, *P* = .0023).

**Figure 4:**
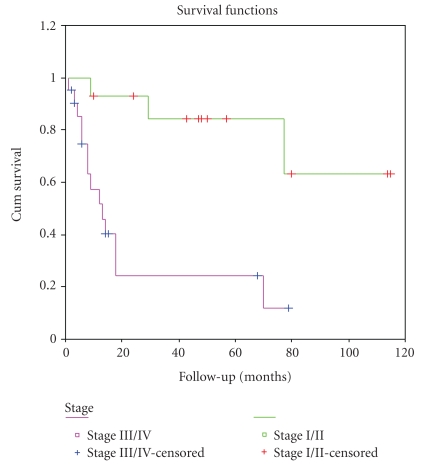
Kaplan-Meier curve based on stage (stage I/II versus stage III/IV, *P* = .003).

**Figure 5:**
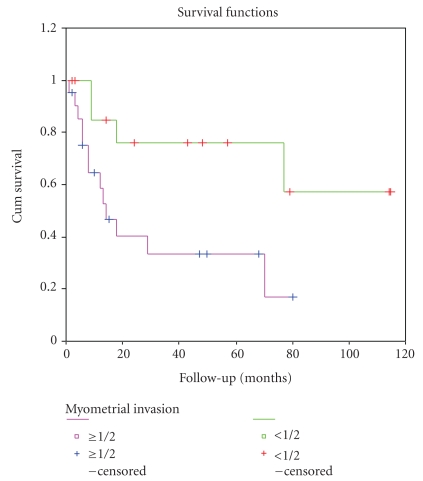
Kaplan-Meier curve based on myometrial invasion (≥1/2 versus <1/2, *P* = .0138).

The results of univariate survival analysis of Her-2/neu protein expression and other clinicopathological factors are shown in Table 3. The 5-year overall survival rate for Her-2/neu IHC positive and negative cases was 12.9% and 68.6%, respectively. Poorer outcome (Kaplan–Meier) was observed for patients with Her-2/neu overexpression positive than negative patients (*P* = .0023) (Figure 3). The 5-year overall survival rate for early and advanced stage cases was 60.4% and 16%, respectively. Advanced stages (*P* = .0006) and deep myometrial invasion (*P* = .0138) were also significantly associtated with a shorter OS (Figures 4 and 5).

## 4. Discussion

UPSC is a rare type of endometrial cancer, whose clinical features are different from endometrioid endometrial adenocarcinoma. The median age of UPSC was 64 years in this study, similar to previous report, but older than the median age of women with endometrioid endometrial cancers in China [25]. Unlike endometrioid endometrial cancers, UPSC was not associated with obesity. The median BWI was 23.8 kg/m^2^ in this study. And most important of all, compared to patients with endometrioid endometrial adenocarcinomas, patients with UPSC tend to have deep myometrial invasion, high-stage diseases, and worse overall survival, as we had discussed previously in [25]. In our study, 61% were late stages. More than half of patients (58.3%) had deep myometrial invasion. The 1-year, 3-year, and 5-year overall survival was 73.1%, 51.9%, and 43.9%, respectively.

Although racial differences in the uterine cancer have been previously published, most of these studies have focused on disparities between African Americans and whites. Santin et al. noted that black patients with UPSC tended to be younger, had higher Her-2/neu expression and short survival than white [26]. Slomovitz [9] summarized the clinical features of 129 cases with UPSC. In his study, 93% patients were Caucasian, and only 2% were Asian. Therefore, little is known about Asian patients with UPSC till now. Recent study had reported the racial disparities between Asians and whites. Zhang et al. [27] compared 2,144 Asians and 32,999 whites with corpus cancer in the United States, and found that Asians presented at a younger age, with more advanced stage disease and higher 5-year survival rates than whites. In our study, similar to whites, main presenting symptoms were abnormal vaginal bleeding and with median BWI of 23.8 kg/m^2^, patients with UPSC ware not associated with obesity. On the other hand, Asian patients with UPSC had younger age (64 years) and more late stage diseases (61%) than whites compared to the previous report [9], in which median age was 68 years for whites and 56% patients were in late stages. The 3-year and 5-year overall survival in our study was 51.9% and 43.9%, respectively, which was a little worse than whites, whose 3-year and 5-year overall survival was 62.6% and 45.9%, respectively [9]. 

Recently, Her-2/neu overexpression was found to be a common event in UPSC. Grushko et al. [28] found that UPSC had more Her-2/neu overexpression than all other types of endometrial cancers (23 of 38, 61% versus 81 of 196, 41%, resp., *P* = .03). Santin et al. [20, 21] reported that 62%~80% of UPSC overexpressed Her-2/neu protein, and others reported overexpression rates varyng from 40% to 48% [29–31]. In contrast, Slomovitz et al. [19] reported overexpression of Her-2/neu in only 18% (12 of 68) of UPSC patients.  Table 4 summmariued the results and methods of the studies on Her-2/neu status in UPSC. In our study, we demonstrated that 36% of patients with UPSC overexpressed Her-2/neu protein. The differences of the rate of Her-2/neu overexpression, might be due to different antibody, inherent intraobserver variability, and most important of all, racial disparity.

In our study, patients with Her-2/neu overexpression were significantly more likely to have advanced stage disease when compared to Her-2/neu negative patients (*P* = .03). Overexpression of Her-2/neu was seen in 50.0% (11/22) of UPSC patients with advanced disease, compared to 14.3% (2/14) of patients with early disease. Similar findings were reported by Slomovitz, Santin, and Díaz-Montes et al., in which overexpression of Her-2/neu in patients with advanced disease were 24%~81.8%, compared to 8%~28.6% of patients with early disease [19, 20, 30]. Díaz-Montes et al. also demonstrated that overexpression of Her-2/neu was associated with higher Ki-67 index, larger tumor sizes, and worse survival outcome.

In survival analysis, we found that the overall survival was worse for patients with Her-2/neu protein positive tumors than Her-2/neu negative ones (*P* = .0023). The 5-year overall survival for Her-2/neu IHC positive and negative cases was 12.9% and 68.6%, respectively. Slomovitz et al. [19] also revealed that the 5-year overall survival in UPSC patients was 0% in Her-2/neu IHC positive cases versus 45% in Her-2/neu negative ones (*P* = .008). And Santin et al. [26] demonstrated that short survival was associated significantly with Her-2/neu overexpression compared with IHC expression 0~1+(*P* = .002). All of these findings suggested that Her-2/neu overexpression was a useful prognostic factor for this aggressive subtype of endometrial cancer. Besides Her-2/neu IHC overexpression, we also found that deep myometrial invasion (*P* = .0138) and late stages (*P* = .003) were associtated with a shorter OS. 

We used CISH to detect the Her-2/neu gene amplification in our study and found Her-2/neu gene amplification in four cases (11.1%). Santin et al. [32] revealed that Her-2/neu gene amplification was correlated with a poor survival outcome in patients with UPSC (*P* = .0084). Because of the small size of Her-2/neu amplification in our study, we did not find the effect of Her-2/neu amplification on overall survival.

Her-2/neu gene amplification was found in all IHC 3+ cases (100%, 3/3). But in IHC 2+ cases, only one out of ten cases had Her-2/neu gene amplification. As we know, patients who have Her-2/neu gene amplification respond better to trastuzumab. Therefore, like breast cancer, the entry criteria of trastuzumab in UPSC should be confined to IHC 3+ cases, excluding the 2+ cases unless it is proved to have Her-2/neu amplification by CISH or FISH. The Gynecologic Oncology Group conducted a phase II study using trastuzumab as a single agent in patients with advanced stage or recurrent endometrial cancer with Her-2/neu IHC 2+ or 3+ staining, but found no activity of trastuzumab in heavily pretreated patients. 13% of cases demonstrated gene amplification and 37% demonstrated moderate or high immunostaining. Entry criteria were then revised to include only those patients whose tumors were FISH positive, but the study was finally closed due to slow enrollment [33]. 

Santin et al. [21] studied the effects of trastuzumab in UPSC cell lines and found that UPSC cell lines were highly sensitive to Herceptin mediated antibody-dependent cellular cytotoxicity (ADCC) and cell proliferation was inhibited. Villella et al. [34] tried to use trastuzumab therapy (4 mg/kg intravenously over 90 min, then maintenance dose of 2 mg/kg intravenously over 30 min weekly until progression of disease) in patients with UPSC who expressed Her-2/neu 3+ by IHC. Two patients with 3+ overexpression received trastuzumab treatment. One patient with IVB disease had complete response and the other with IIIC disease had stable disease for 3 months. Targeting Her-2/neu might be beneficial for a select group of patients with UPSC.

In conclusion, our data revealed that Chinese patients with UPSC had characteristics of deep myometrial invasion, advanced stages and poor overall survival. Her-2/neu overexpression, rather than Her/neu gene amplification, is associated with advanced stage and poor survival outcome. Her-2/neu might be a therapeutic target in refractory UPSC patients, but it should be further evaluated by randomized clinical trials.

## Figures and Tables

**Table 1:** Clinicopathological features by Her-2/neu IHC status.

	Clinicopathological features by Her-2 IHC status		
	positive (*n* = 13)	negative (*n* = 23)	*P* value
Age (years)			
Median (range)	64 (58–77)	61 (45–81)	.22
Stage			.03
I~II	2 (15.4%)	12 (52.2%)	
III~IV	11 (84.6%)	11 (47.8%)	
Myometrial invasion			.78
<1/2	5 (38.5%)	10 (43.5%)	
*⩾*1/2	8 (61.5%)	13 (56.5%)	
Histology			.33
Pure	9 (69.2%)	12 (52.2%)	
Mixed	4 (30.8%)	11 (47.8%)	
LVSI			.4
Absent	7 (53.8%)	9 (39.1%)	
Present	6 (46.2%)	14 (60.9%)	
Grade			.45
II	3 (23.1%)	3 (13.0%)	
III	10 (76.9%)	20 (87.0%)	
p53			.08
Absent	1 (7.7%)	8 (34.8%)	
Present	12 (92.3%)	15 (65.2%)	
ER			.69
Absent	10 (76.9%)	16 (69.6%)	
Present	3 (23.1%)	7 (30.4%)	
PR			.66
Absent	11 (84.6%)	18 (78.3%)	
Present	2 (15.4%)	5 (21.7%)	

**Table 2:** Correlation of Her-2/neu status by IHC and CISH.

		Her-2 protein expression by IHC				*P *value
		3 + (*n* = 3)	2+ (*n* = 10)	1+ (*n* = 11)	0 (*n* = 12)	
Her-2 Amplification by CISH	High-level	2	1	0	0	.001
	Low-level	1	0	0	0	
	No	0	9	11	12	

**Table 3:** Univariate analysis of Her-2/neu IHC status and clinicopathological factors with overall survival.

	No. of patients	No. of Deaths	Survival Rate (%)			*P* value
			1-year	3-years	5-years	
	36	17	73.1	51.9	43.9	
Her-2/neu by IHC						.0023
Positive	13	11	60	25.7	12.9	
Negative	23	6	81	68.6	68.6	
Stage						.0006
I~II	14	3	92.6	84.5	60.4	
III~IV	22	14	60	26.7	16	
Myometrial invasion						.0138
<1/2	15	4	85.7	77.6	55.4	
*⩾*1/2	21	13	64.1	33.1	19.9	
Histology						.948
Pure	21	10	74.4	50.6	30.3	
Mixed	15	7	71.4	54	36	
LVSI						.4884
Present	20	10	63.2	51.1	30.7	
Absent	16	7	86.2	52.8	37.7	
Grade						.8689
II	6	3	75	50	50	
III	30	14	68.4	51.8	41.5	

**Table 4:** Summary of related articles on Her-2/neu status in UPSC.

Year	Author	Methods	Number of Patients	Positivity (%)
1994	Prat et al.	IHC	10	40% (4/10)
2001	Halperin et al.	IHC	22	45% (10/22)
2002	Santin et al.	IHC	10	80% (8/10)
2004	Slomovitiz et al.	IHC	68	18% (12/68)
		FISH	12	17% (2/12)
2005	Santin et al.	IHC	26	62% (16/26)
		FISH		42% (11/26)
2006	Diaz-Montes	IHC	25	48% (12/25)
2008	Grushko et al.	IHC	38	61% (23/38)
		FISH	28	21% (6/28)
